# Coccidioidomycosis: a growing global concern

**DOI:** 10.1093/jac/dkaf002

**Published:** 2025-03-14

**Authors:** Fariba M Donovan, Omar Marín Fernández, Gurjinder Bains, Lisa DiPompo

**Affiliations:** Valley Fever Center for Excellence, University of Arizona College of Medicine—Tucson, Tucson, AZ, USA; Division of Infectious Diseases, Department of Medicine, University of Arizona College of Medicine—Tucson, Tucson, AZ, USA; BIO5 Institute, University of Arizona, Tucson, AZ, USA; F2G Ltd, Manchester, UK; Shionogi B.V., London, UK; F2G Inc., Princeton, NJ, USA

## Abstract

Coccidioidomycosis (CM) has been a recognized disease for about 130 years. The organisms (*Coccidioides* spp. fungi) inhabit desert soil in the southwestern USA, Mexico, and parts of Central and South America. Natural events such as dust storms, wildfires or outdoor activities including construction and gardening can disrupt the fungal arthroconidia, which easily become airborne and inhaled by the host. Approximately 60% of those exposed to arthroconidia are asymptomatic and do not require medical attention, but 30% show signs of pulmonary infection with symptoms ranging from a flu-like illness to pneumonia. In 5%–10% of cases serious or disseminated disease develops, which requires prompt diagnosis and management. About 1%–3% of infections disseminate to the CNS and if not appropriately treated are often fatal. There is an urgent need for improved diagnostics and treatments.

## Introduction

Coccidioidomycosis (CM), also known as Valley fever (VF), is caused by the dimorphic fungi *Coccidioides* spp. A broadened awareness of this potentially life-threatening disease is becoming increasingly important for healthcare providers worldwide. The purpose of this article is to highlight recent updates in epidemiology, clinical presentations, diagnosis, diagnostic challenges and available CM treatment/prevention. CM was first described by Alejandro Posadas in 1892.^1^ Historically it has been considered endemic to the arid deserts of California, Arizona and Mexico. Studies have demonstrated an expanding range of the fungus to other areas such as Utah, Texas and Washington state, possibly due to climate change and population growth.^2^ In addition to the USA, *Coccidioides* spp. exist in other countries, including Mexico in the areas of Sonora, Nayarit, Jalisco and Michoacán, as well as Coahuila, Durango and San Luis Potosi. It is also reported in Central America (Guatemala, Honduras and Nicaragua), and South America (Argentina, Bolivia, Paraguay, Venezuela, Colombia and Brazil).^3^ Migration and tourism are raising concerns that CM will grow beyond a regional disease to a global problem.^4^ The fungus can survive indefinitely just below the soil surface as mycelia (saprobic phase). Growth is favoured during the rainy season, and soil disruption during dry, warm weather promotes the release of spores (arthroconidia).^5^ Upon inhalation, arthroconidia reach the host terminal bronchioles and alveoli, beginning the disease process (parasitic phase). The initial pathogen–host interaction is not well understood and is an area of intense current research.^6,7^

In humans, most cases (60%) are self-limited pulmonary infections that in many instances are mistaken for community-acquired pneumonia (CAP).^8^ Some individuals develop more serious pulmonary complications such as cavitary pneumonia (30%).^9,10^ The remaining 10% develop a complicated or severe disease.^11^ Up to one-third of this group will develop disseminated disease with life-threatening sequelae such as meningitis and spinal abscesses.^12^ The genetic basis of such a wide disparity of clinical presentations is yet to be elucidated.^13^ Nonetheless, some of this disparity could be explained by host immune response variability and resulting collateral tissue damage secondary to specific coccidioidal antigens.^14–16^

Challenges remain in early CM diagnosis. For instance, CM can mimic the clinical presentation of diseases such as CAP, influenza, coronavirus disease (COVID-19) and rheumatoid arthritis. Even in endemic areas there is a gap in healthcare provider CM awareness.^17^ This is thought to be related to the large number of practitioners in endemic areas who were trained elsewhere. Another important challenge is overcoming deficiencies in current CM serological testing, such as delayed turnaround time as well as limited sensitivity and specificity. With an expanding use of biological response modifiers and other immune modulating agents there is a critical need to develop a test for CM immunity.^18^

There are also unmet needs for better CM treatment and prevention. Notably, despite the IDSA guideline recommendations for CM treatment with fluconazole (and other azoles), this medication class has not been approved by the FDA for this indication.^19^ The development of a fungicidal medication with a shorter treatment duration, greater tolerability and less toxicity remains a priority. In addition, development, introduction and acceptance of a vaccine for CM prevention is an ongoing challenge, especially for at-risk populations. The goal of this article is to heighten global CM awareness and spark further interest in related research for the improved diagnosis, treatment and prevention of this fungal disease.

## Epidemiology

The two known *Coccidioides* species are *C. immitis* and *C. posadasii*. *C. immitis* is found predominantly in California, and can extend to Baja California, Arizona as well as parts of Utah and Washington state. *C. posadasii* is found predominantly in Arizona and can range to Utah, New Mexico, Texas, Mexico and parts of Central and South America.^20–22^ Over the past few decades there have been increasing reports of infections due to endemic fungi in areas thought to be non-endemic.^23^ Several cases of CM have been reported in individuals who acquired the infection in eastern Washington state.^24^

A total of 20 003 cases were reported to CDC in 2019, but this is believed to greatly underestimate the actual number of CM cases. This is thought to be secondary to CM not being reportable in all states, along with the aforementioned gap in healthcare provider awareness and the low sensitivity of CM screening serologies.^25,26^ In addition, there are rare reports of infections imported into countries across Europe.^4,27,28^

There are numerous possible reasons for these shifts in geography and disease frequency such as increased use of immune suppressive medications, more diagnostic tests, and global factors such as migration, increased travel and climate change. The CDC VF maps clearly demonstrate the expanded areas where known or suspected CM cases have been identified. The aforementioned factors and an expanding range make it imperative healthcare practitioners obtain a comprehensive travel history to recognize CM and prevent diagnostic delay and increased morbidity.^17^ Numerous studies have confirmed populations at risk for progressive CM including those of African ethnicity, Filipinos, pregnant women and the immunocompromised.^29–31^

It should be emphasized that unlike opportunistic fungal infections, severe CM is not limited to immunocompromised hosts, and most patients with disseminated CM (DCM) have no identifiable immune deficiencies. Nonetheless, awareness of the aforementioned populations will remain important and a focus as researchers discover specific gene mutations and their products that could identify individuals at risk for DCM.^32^ Efforts and success in this regard could lead to earlier interventions such as targeted vaccine strategies and treatments resulting in fewer disease complications and improved antimicrobial stewardship.

## Life cycle

*Coccidioides* is a dimorphic fungus that alternates between saprobic (mycelia) and parasitic (arthroconidia/spherule/endospore) phases. Under certain environmental conditions the fungus can cycle through the mycelial phase without infecting a mammalian host.^33,34^ Nonetheless, the molecular mechanisms that initiate the morphological switch from a saprobic to a parasitic phase are not well understood.^35^

In the saprobic phase, *Coccidioides* transitions from mycelium to arthroconidia. Arthroconidia are released and become airborne by soil disturbance. Widespread events such as wildfires, and even more limited activities like farm work, construction projects or simple gardening can lead to arthroconidia inhalation by the host resulting in CM.

**Figure 1.**
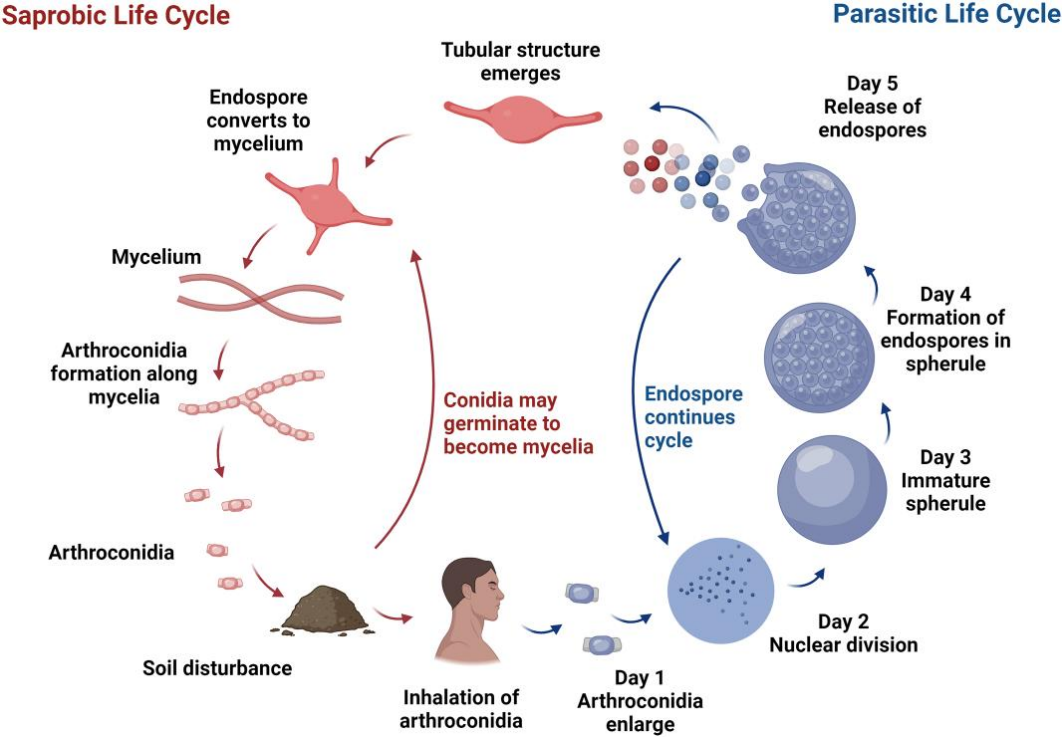
Life cycle of *Coccidioides*. Both *Coccidioides* species share the same asexual life cycle, switching between saprobic (left) and parasitic (right) life stages. The saprobic cycle is found in the environment and produces the infectious arthroconidia. The conidia may be inhaled by a susceptible host or may return to the environment to continue the saprobic life cycle. Image used under Creative Commons Licence. Lewis ERG, Bowers JR, Barker BM. Dust devil: the life and times of the fungus that causes valley fever. *PLoS Pathog* 2015; **11**(5): e1004762. doi:10.1371/journal.ppat.1004762.

For humans, a minimum inoculum is unknown, but it has been stated that the infectious dose can be just a single arthroconidium.^36^ Arthroconidia are small (4 µm) barrel-shaped yeasts that can reach the terminal bronchioles and alveoli. Almost immediately they will transform into large spherules, which over a few days can rupture to release endospores. If this process is not controlled by the host’s innate immune system, it can repeat itself exponentially (Figure 1).

## Clinical presentations

Approximately 60% of people who inhale *Coccidioides* arthroconidia develop infections that are subclinical with either mild symptoms or none at all. Most of the remainder (approximately 30%) experience a respiratory syndrome of CAP including a non-productive cough, chest pain and dyspnoea.^8,12^ Signs and symptoms of fever, night sweats, fatigue and weight loss are frequent.^11^ Also, without any respiratory complaints, patients may develop generalized immunologically mediated complaints of arthralgias, myalgias or skin rashes—so much so that a synonym for CM is ‘desert rheumatism’.^37^ Eventually, despite often protracted morbidity, most patients resolve their illness whether or not they are treated with antifungal medications. Some will have a residual asymptomatic pulmonary nodule evident on chest imaging.^38^ The remainder (approximately 10%) will develop either fibro-cavitary pulmonary lesions or haematogenous spread to non-CNS sites such as bone or skin, and to CNS sites. The most frequently involved CNS sites are the meninges. These complications require life-long medical management. Fortunately, those with self-limited, and possibly even those with complicated courses of their first infection, rarely if ever develop an illness from a second exposure to *Coccidioides.*^11^

For the purpose of this article, we are classifying the disease presentations into four distinct entities including acute pulmonary CM, chronic pulmonary CM, non-CNS DCM and CNS DCM, although there is debate among the experts whether CM is four separate disease states or a continuous spectrum of mild to very severe disease.

### Acute pulmonary CM

Symptoms of acute pulmonary CM are thought to appear from 1 to 3 weeks after exposure (an average of 2 weeks).^37^ The symptoms include fever, chest pain, cough, malaise, anorexia, headache, pharyngitis, chills, joint manifestations, erythema nodosum, conjunctivitis, erythema multiforme and urticaria.^39^ Because of a similar presentation to CAP, influenza and even COVID-19, the diagnosis often is delayed, even in endemic areas.^17,40–42^ This diagnostic delay leads to unnecessary use of antibacterials, laboratory tests, imaging and invasive procedures. All of which contribute to adverse health consequences and additional costs.^17^

### Chronic pulmonary CM

In approximately 4% of patients with coccidioidal pneumonia, infiltrates do not completely resolve and result in a chronic pulmonary process. Patients with underlying lung disease, diabetes mellitus or immunosuppression are more likely to develop this complication.^43,44^ Chronic pulmonary CM includes pulmonary nodules and persistent fibro-cavitary disease. Pulmonary nodules often are found incidentally and can be confused with lung cancer. Fibro-cavitary lesions likely develop from liquefied pulmonary nodules.^45^ A small proportion of cavities will rupture leading to an abrupt onset of dyspnoea and chest pain. If a ruptured coccidioidal cavity is diagnosed, the preferred therapy is prompt surgical resection as definitive or adjunctive treatment to antifungal treatment.^46^ Proximally located cavities may require a lobectomy due to the surrounding inflammation, and decortication to facilitate re-expansion of the remaining lung.

In general, patients with fibro-cavitary CM are often symptomatic with night sweats, fatigue and weight loss, in addition to pulmonary symptoms, such as chest pain, cough, haemoptysis and sputum production. Radiographically, the changes can be quite extensive and often include a complex mixture of infiltrates, fibrosis and cavitation.^47^

### Non-CNS DCM

When coccidioidal infection spreads beyond the lungs, it usually does so in the first several months after initial infection and nearly always in the first year.^11^ There are significant mortality differences between various forms of DCM.^48^ CNS dissemination has the highest mortality rate; however, non-CNS dissemination also exhibits an unacceptably high morbidity and mortality.^48^

The most common sites of non-CNS dissemination are skin, subcutaneous tissues and vertebral column; however, virtually any part of the body can be affected.^11^ Due to such a broad spectrum of presenting signs and symptoms clinicians should keep non-CNS DCM in the differential diagnosis when evaluating patients who reside or have visited endemic areas.^49^ Cutaneous granulomatous lesions, which could be mistaken for skin cancer or other infections, represent the most benign form of coccidioidal dissemination. Subcutaneous soft tissue abscesses are often diagnosed as bacterial infections and can be quite complicated. Osteomyelitis can masquerade as a soft tissue abscess and often requires needle aspiration or incision of larger lesions as both a diagnostic and treatment modality. Non-CNS DCM can mimic lymphadenitis, peritonitis, epididymitis, prostatitis and septic arthritis. Commonly infected joints are the knees, wrists and ankles. Patients present with the typical symptoms of pain, limited range of motion, swelling and effusion. Most concerning is osteomyelitis especially when it involves the vertebral column, with extension to the adjoining soft tissues in the form of paraspinal or epidural abscesses.^16,19^ Even with the use of current antifungal treatments, some patients are at risk of spinal cord compromise, paralysis or even death.

### CNS DCM

*Coccidioides* can spread via the lymphatic system or bloodstream to the CNS, especially the basilar meninges, within weeks to months following the initial respiratory infection.^50^ Untreated meningitis is nearly always fatal, therefore early diagnosis and initiation of therapy is crucial. Patients with CNS dissemination predominantly present with headaches that are consistent, persistent and progressive.^51–53^ Patients often complain of ‘feeling off mentally’ and have unexplained nausea and vomiting. Hydrocephalus is the most common complication of CNS coccidioidal infection and is identified by enlarged ventricles via brain imaging. Approximately 40% of individuals with CNS involvement will have hydrocephalus at presentation or will develop it during their disease course. Besides the symptoms noted above, patients with hydrocephalus often complain of altered sensorium, a gait abnormality and urine incontinence. Some patients with coccidioidal meningitis and hydrocephalus remain asymptomatic but require ongoing medical treatment and serial imaging every 3–6 months until clinical stability has been achieved. Symptomatic patients with increased intracranial pressure require placement of a ventriculoperitoneal (VP) shunt by neurosurgery.

## Diagnosis

In addition to patient history and clinical presentations, CM diagnosis relies upon basic laboratory tests such as complete blood count, erythrocyte sedimentation rate, eosinophil count and chest X-ray. Although CM diagnosis with fungal culture or histopathology is definitive, it remains labour intensive, invasive and expensive. Therefore, serological testing remains the mainstay of CM diagnosis. Currently available diagnostic testing has variable accuracy, particularly in certain patient populations, and new tests may offer improved accuracy. If CM is suspected initial testing is performed using antibody enzyme immunoassays (EIAs). The EIA tests for coccidioidal IgM/IgG (sensitivity 59%–88%, specificity 68%–96%). Positive EIA will trigger confirmatory immunodiffusion (ID) (sensitivity 60%, specificity 99%) and reflex to complement fixation (CF) (sensitivity 65%–83%, specificity 98%).^54^ A positive coccidioidal serology mostly confirms the CM diagnosis but does not completely exclude other aetiologies. Conversely, negative CM serology does not entirely exclude CM.^11,19^ EIA is simpler to perform and provides results the same day, whereas ID and CF are more complex and require 2 to 6 days to provide results.^55^ Despite their use as screening tests, the performance characteristics of EIAs remain unclear.

These limitations have prompted the development of a coccidioidal antigen test to identify the fungal antigen in blood, urine and CSF. Combining *Coccidioides* antigen testing with standard antibody tests for CM diagnosis has shown additional benefits in immunocompromised patients.^56^*Coccidioides* urine antigen test is used when antibody testing is negative, but CM diagnosis is suspected in an immunocompromised host.^57^ Another use of antigen testing is in the measurement of coccidioidal antigen in CSF as an indicator for the response to meningitis treatment.^58^

The need to improve the availability, ease of use, rapidity, reliability and cost has sparked refinement of a lateral flow assay (LFA). LFAs would be invaluable as readily available, affordable point-of-care tests that will speed the CM diagnosis and lessen antibiotic overuse.^59^ For instance, a recent study suggested combining LFA with procalcitonin measurement could reduce unnecessary antibacterial use by 77%.^59^

Another tool that has gained attention in CM diagnostics is real-time PCR.^60^ This PCR test has the advantage of a 4 h turnaround time and a high sensitivity and specificity for specimens obtained from bronchoalveolar lavage (BAL) or bronchial wash (BW). A notable disadvantage for PCR use is its lower sensitivity and specificity in other tissue specimens besides BAL and BW.^61–63^ Another potential diagnostic tool for early CM detection is represented by the ongoing development of breath tests for fungal and/or host volatile metabolites as biomarkers for coccidioidal infection.^64^ The wide variety of these potential diagnostic test strategies underscores the need for and importance of early CM diagnosis.

## Treatment

The therapeutic approach for CM patients is undergoing continuous evaluation and refinement. The decision of who and when to treat for CM depends on several factors. Foremost is the disease severity and health status of the patient, including comorbidities and immune status. Because 60% of those who acquire *Coccidioides* are asymptomatic and over 30% may have a limited pulmonary syndrome, recent treatment recommendations advise observation in otherwise healthy or recovering patients and reserve antifungal treatment for those with severe disease, persistent symptoms or the immunocompromised.^12,19,65^ The IDSA guidelines recommend education, close observation and supportive care in those patients with uncomplicated CM pneumonia and asymptomatic CM pneumonia with cavity.^19^

Antifungal medications and/or surgery are reserved for severely symptomatic CM pneumonia with or without cavities. The recommended treatment for this group is fluconazole or itraconazole. Due to the fungistatic nature of these medications, even with a ‘successful’ short-term treatment, it is possible these patients could develop recurrence and/or dissemination after discontinuation.^66^

The DCM treatment guidelines include high-dose fluconazole or other azoles with or without lipid formulation of amphotericin B.^19,67^ These recommendations are for treatment of extrapulmonary soft tissue infection as well as vertebral osteomyelitis and coccidioidal meningitis.^12,19^ As previously mentioned, surgical debridement or VP shunt placement may be necessary with concurrent antifungal treatment in certain clinical settings. The recommended treatment duration for disseminated disease is variable. Treatment is at least 1 year for non-CNS dissemination.

For CNS disease treatment is currently lifelong, as azole therapy suppresses rather than cures the disease. The recommendations for lifelong azole therapy include treatment even for those who achieve remission and do not deteriorate. Clinical response is monitored by resolution of symptoms such as fever and headache along with a reduction in CF antibody titre. Symptom recurrence in those successfully treated for non-CNS disease should prompt repeat of CF titres and/or imaging and possible reintroduction of antifungals.

As mentioned, the mainstay of CM treatment is the triazole class, specifically fluconazole and itraconazole, but there are currently no randomized controlled trials addressing CM treatment. Greater experience in the use of these agents, medication costs, adverse event profiles, drug absorption and lack of therapeutic drug monitoring requirements, have prompted azole use as primary CM treatment. Fluconazole is typically chosen as initial treatment over itraconazole as it is less toxic, has fewer drug–drug interactions and requires fewer pharmacokinetic (PK) considerations when dosing.^68^ Other azoles such as posaconazole, voriconazole and isavuconazole are reserved for patients who are either intolerant or not responding to fluconazole or itraconazole.^19^ Use of the lipid formulation of amphotericin B is reserved for seriously ill patients and is limited by its adverse side effects including nephrotoxicity, anaemia and fever (Table 1).^69,70^ The intrathecal administration of amphotericin B deoxycholate is reserved for intractable CNS-DCM in a few centres with expertise to perform the procedure.^71^

**Table 1.** Adverse effects of the antifungals used to treat coccidioidomycosis^a^

Adverse drug effects	Fluconazole	Itraconazole	Voriconazole	Posaconazole	Isavuconazole	AMB^b^
Blood and lymphatic system	+++/++	++++/+++	++++/++	++++/++	+++	+++
Hypersensitivity reactions	++	++++	+++	++	+++	+
Metabolism and nutrition disorders	+++/++	++++/+++		++++/+++	++++	
Electrolyte imbalance	++	++++/+++	++++	++++	++++	+++++
Renal toxicity	±	±	±	±	±	+
Hyperglycaemia		++++		+++		++++
Psychiatric disorders	+++	++++	++++	+++/++	++++/+++	
Myositis and neuropathies		++++	+++	+++	+++	+
Cardiac side effects	++	++++/+++	++++/++	+++/++	+++	++++/+
Gastrointestinal disorders	++++	+++++/++++	+++++/++	+++++/++	++++/+++	+++++
Pancreatitis		+	+++	+++		
Hepatotoxicity	+++++	++++	++++	++++	++++/+++	++
Skin (rash and other)	++++/++ (SJS, DRESS)	+++++/+ (SJS)	+++++/+ (SJS, SCC, DRESS)	++++/++	++++/+++	++++
Hormone disruption			+++/++	++		
Bone pain	++		++	+++		
Vascular disorders			++++/+++	++++/++	++++/+++	++++
Visual disorders			+++++/++	+++/++		

+++/++ uncommon versus rare. AMB, amphotericin B; DRESS, drug reaction with eosinophilia and systemic symptoms; SCC, squamous cell carcinoma; SJS, Stevens–Johnson syndrome.

^a^Very common (+++++), common (++++), uncommon (+++), rare (++), reported but frequency not known (+), conflicting reports (±). Blank boxes indicate not reported in summary of product characteristics.

^b^Fluconazole, itraconazole, voriconazole, posaconazole, isavuconazole, and lipid formulation of AMB.^72,75–79^

The azoles share some similar adverse events including hepatotoxicity, cardiac disorders and serious dermatological reactions such as Stevens–Johnson syndrome (SJS) (Table 1).^72–74^ The adverse side effects of fluconazole, the most commonly prescribed medication, include alopecia, cheilitis, dry skin and QT interval prolongation. The adverse effects of voriconazole include serious dermatological reactions including phototoxicity and squamous cell carcinoma risk, which requires avoidance of direct sunlight. Life-threatening dermatological conditions have also been reported such as SJS, toxic epidermal necrolysis, and drug reaction with eosinophilia and systemic symptoms (DRESS). Other important reported adverse effects include visual impairment and visual hallucinations.^75^ Posaconazole also carries risk of cardiac side effects similar to voriconazole, including QT interval prolongation and more rarely torsade de pointes.^76^ Posaconazole can induce pseudohyperaldosteronism manifesting as hypertension and hypokalaemia when trough levels are >3.5 μg/mL.^77^ Other reported adverse effects include SJS, visual disturbances and psychiatric disorders. Isavuconazole is generally well tolerated but unfortunately has a high cost and, in cases of salvage treatment for chronic pulmonary fungal diseases, could cause CNS side effects such as risk of delirium.^78–80^ Both posaconazole and voriconazole often require therapeutic drug monitoring, as they have variable or non-linear PK respectively. It should be noted that all azoles have significant potential drug interactions that can lead to toxic blood levels of many other medications. Conversely, the presence of a concomitant perpetrator drug can increase or decrease azole levels to a supra- or sub-therapeutic range.^68^

These limitations are prompting the investigation of novel antifungals for CM that would have a shorter treatment duration and lead to an improved quality of life. For instance, the ideal agent would be fungicidal. It would also have both oral and IV preparations, excellent absorption/bioavailability, good tissue concentrations especially in CSF, and minimal side effects. One older and several newer agents are being investigated including nikkomycin Z, ibrexafungerp, oteseconazole, fosmanogepix, olorofim and SUBA (SUper BioAvailability)-itraconazole (Table 2).^81^ To date nikkomycin Z and olorofim are the most investigated. Nikkomycin Z, a chitin synthase inhibitor discovered in 1976, has shown promise in animal models of pulmonary or brain infection. Unfortunately, its development has been limited primarily by a lack of funding.^82,83^ A new medication under investigation for CM treatment is olorofim.^84^ It inhibits de novo pyrimidine biosynthesis by preventing the catalytic activity of dihydroorotate dehydrogenase (DHODH).^84,85^ It is being considered for treatment of dimorphic and filamentous fungi including *Coccidioides.*^85^ It is currently being investigated for refractory DCM in humans, after being demonstrated to be fungicidal in the treatment of DCM in an animal model.^86^

**Table 2.** Antifungals in development with activity against *Coccidioides* spp.

Drug	Class	Mode of action	Spectrum of activity	Stage of development	Route of admin	Active gov identifier
Nikkomycin Z	Chitin synthase inhibitor	Cell-wall synthesis inhibitor	*Candida*, *Aspergillus*	Phase I completed, lack of funding and volunteers caused the termination of Phase II studies	Oral	NCT 00834184
Ibrexafungerp	Triterpenoid	Glucan synthase inhibition	*Candida*, *Aspergillus*, rare moulds, dimorphic fungi	Phase III, FDA-approved for vulvovaginal candidiasis	Oral, IV	NCT 03059992
Osteconazole^a^	Tetrazole	Lanosterol 14α-demethylase inhibitor	*Candida*, *Cryptococcus*	Phase III completed; approved for recurrent vulvovaginal candidiasis	Oral	NCT 03562156 NCT 03561701
Fosmanogepix	First in class, Gwt1 inhibitor	Cell wall-linked mannoprotein inhibition	*Candida*, *Aspergillus*, rare moulds, dimorphic fungi	Phase II completed; received fast-track status for IV and oral formulations	Oral, IV	NCT 04240886
Olorofim	Orotomide	DHODH inhibitor	*Aspergillus*, rare moulds, dimorphic fungi	Phase II completed; Phase III recruiting; FDA breakthrough therapy designation	Oral	NCT 03583164 NCT 05101187
SUBA-itraconazole	Triazole	Ergosterol synthesis inhibition	*Candida*, *Aspergillus*, dimorphic fungi	FDA-approved (2018) for the treatment of aspergillosis, histoplasmosis and blastomycosis (patients with AMB contraindications)	Oral	NCT 03572049

Admin, administration; AMB, amphotericin B; DHODH, dihydroorotate dehydrogenase; Gwt1, glycosylphosphatidylinositol-anchored wall protein transfer 1; SUBA, SUper BioAvailability-itraconazole.

^a^ *In vitro* antifungal activity against *Coccidioides* spp. and efficacy in treating both respiratory and CNS infections in lethal murine models of this disease.

Ibrexafungerp (Brexafemme^®^) is a first-in-class oral triterpenoid that exerts its activity via glucan synthase inhibition. It was recently FDA-approved for the treatment and recurrence of vulvovaginal candidiasis. It is in Phase III clinical trials for the potential treatment of invasive candidiasis.^81^ It exhibits *in vitro* and *in vivo* efficacy against *Coccididoides* spp. and is under development consideration for CM.^87,88^ Oteseconazole (Vivjoa^®^) is a newly approved orally bioavailable azole and selective inhibitor of fungal cytochrome P450 enzyme 51 (CYP51) that has demonstrated efficacy against *Coccidioides* spp. *in vitro* and in animal models.^89^ Nonetheless, development efforts have focused on activity against yeasts and other moulds such as the reduction of recurrent vulvovaginal candidiasis. Fosmanogepix is a first-in-class antifungal with a novel mechanism of action. It is the *N*-phosphonooxymethylene prodrug of manogepix, an inhibitor of the fungal enzyme Gwt1 (glycosylphosphatidylinositol-anchored wall protein transfer 1). It has been demonstrated to be efficacious *in vivo* for mouse models of disseminated *C. immitis.*^90^ Its development is focused on Phase II clinical trials for the treatment of invasive fungal infections caused by *Candida*, *Aspergillus* and rare moulds.^91^ SUBA-itraconazole (Tolsura^®^) is a new formulation of itraconazole using SUBA, a nanotechnology developed to improve oral bioavailability. A prospective, multi-centre, randomized, open-label, parallel arm study comparing SUBA-itraconazole with conventional itraconazole has been completed including CM adult patients with proven or probable invasive endemic fungal infections. Outcome measures for the study include PK, safety, efficacy, tolerability and health economics.^92^ Table 3 details the *in vitro* activity of some of the new antifungals against *Coccidioides.*^86,89,93–95^

**Table 3.** Activity of newer antifungal drugs against *Coccidioides* spp.^a^

Drug	Species	*N*	Range (MIC)	MIC_50_/MEC_50_	MIC_90_/MEC_90_	GM MIC/MEC	Reference
Olorofim	*C. immitis*	21	≤0.008–0.015	≤0.008	0.015	0.009	86
	*C. posadasii*	24	≤0.008–0.015	≤0.008	0.015	0.009	
	*Coccidioides* spp.^b^	202	≤0.008–0.5	0.008	0.015	0.010	93
Oteseconazole	*C. immitis*	23	1–4	2	2	1.480	89
	*C. posadasii*	29	1–4	1	2	1.430	
VT-1598	*C. immitis*	1	0.5	NA			94
	*C. posadasii*	1	1				
	*Coccidioides* spp.^c^	40	≤0.5	ND		0.217	95
	*C. immitis*	U	U			0.180	
	*C. posadasii*	U	U			0.250	
Manogepix	*C. immitis*	5	0.001–0.004	ND	0.008	0.004	90
	*C. posadasii*	5	0.004–0.015				

GM, geometric mean; MEC, minimum effective concentration; N, number of each fungal strain; NA, not applicable; ND, not determined; U, unknown.

^a^MICs are presented for olorofim, oteseconazole and VT-1598 and generally represent the lowest drug concentration that gave ≥80% inhibition of growth. MECs are presented for manogepix and represent the lowest drug concentration that uniformly shortened hyphal formation. All values are presented in µg/mL.

^b^MIC determined as lowest concentration that gave 100% inhibition of growth compared with the drug-free control.

^c^Data encompass two rows below, included to demonstrate differences in geometric mean between two species.

With the expanding burden of CM, clinicians treating these patients are hopeful industry or government partnerships and support will result in successful clinical trials of these antifungals or future treatments.

## Prevention

It should be noted that human to human transmission of CM is extremely rare and therefore disease prevention is focused on two areas: environmental exposure prevention and vaccine development. In endemic areas, the National Institute of Occupational Safety and Health (NIOSH) recommends workers at risk of CM exposure consider strategies such as use of N95 masks, air-conditioned/HEPA-filtered (heavy equipment) cabs and tamping soils with water to prevent fungal exposure.^96^ For non-occupational exposure prevention, it is recommended that individuals avoid close contact with dust or dirt including yard work, gardening and digging.^25^ The IDSA guidelines for the immunocompromised recommend active surveillance for CM symptoms and frequent serology, and reserve prophylactic fluconazole for patients undergoing organ transplantation.^19^ Both the CDC and IDSA provide guidance for laboratory personnel for exposure prevention and require *Coccidioides* handling for research to be done in a biosafety level 3 facility.^97^

Currently there are a number of CM vaccine candidates being considered and each has its own advantages.^98^ The first is a live, attenuated vaccine that has been tested and proven to be effective in a canine model.^99^ It has the advantages of potentially being developed at a reasonable cost and is further along in the approval process. The second is a recombinant vaccine that is a construct of coccidioidal wall antigens and adjuvant that, despite being in an earlier developmental phase, potentially has fewer associated risks than a live vaccine. A newer candidate is being developed based upon the experience with COVID-19 vaccines and uses RNA/DNA vaccine platforms.^100,101^ Its development promises to broaden the understanding of early fungal–host interaction in the non-human primate model. Ultimately all CM vaccine candidates face similar hurdles in terms of substantial development costs, and public acceptance.

## Conclusions

There is a growing concern about the expanding geographical range, patient morbidity/mortality and increasing economic burden associated with CM. Similarly, there is an expanding immunocompromised population at risk secondary to either their disease or to treatment with immunosuppressant agents, both among residents and visitors to CM endemic regions. Although this is likely the same for other fungi, CM stands apart for its invasive ability and adverse effects in otherwise healthy individuals. For healthcare providers and CM researchers the most prominent challenges remain greater public awareness, healthcare education, improved early diagnostic methods, vaccine development and improved therapeutics. In this regard the introduction of a fungicidal, well-tolerated, limited treatment course medication remains a critical but attainable goal in the near future.
